# RETRACTION: CDKL5 Promotes Proliferation, Migration, and Chemotherapeutic Drug Resistance of Glioma Cells via Activation of the PI3K/AKT Signaling Pathway

**DOI:** 10.1002/2211-5463.13946

**Published:** 2024-12-06

## Abstract

**RETRACTION**: Z. Jiang, T. Gong, and H. Wei, “CDKL5 Promotes Proliferation, Migration, and Chemotherapeutic Drug Resistance of Glioma Cells via Activation of the PI3K/AKT Signaling Pathway,” *FEBS Open Bio* 10, no. 2 (2020): 268‐277, https://doi.org/10.1002/2211‐5463.12780.

The above article, published online on 21 January 2020, in Wiley Online Library (wileyonlinelibrary.com), has been retracted by agreement between the journal Editor‐in‐Chief, Miguel A. De la Rosa; FEBS Press; and John Wiley and Sons Ltd. The retraction has been agreed upon following an investigation into concerns raised by a third party, which revealed inappropriate image duplications between this (Figure 3E, 4A and E, 5B and E) and other articles that were previously published or published in the same or following year. Given the extent of the identified issues, the editors have lost confidence in the data presented and consider the conclusions of this manuscript substantially compromised.

**RETRACTION**: Z. Jiang, T. Gong, and H. Wei, “CDKL5 Promotes Proliferation, Migration, and Chemotherapeutic Drug Resistance of Glioma Cells via Activation of the PI3K/AKT Signaling Pathway,” *FEBS Open Bio* 10, no. 2 (2020): 268‐277, https://doi.org/10.1002/2211‐5463.12780.

The above article, published online on 21 January 2020, in Wiley Online Library (wileyonlinelibrary.com), has been retracted by agreement between the journal Editor‐in‐Chief, Miguel A. De la Rosa; FEBS Press; and John Wiley and Sons Ltd. The retraction has been agreed upon following an investigation into concerns raised by a third party, which revealed inappropriate image duplications between this (Figure 3E, 4A and E, 5B and E) and other articles that were previously published or published in the same or following year. Given the extent of the identified issues, the editors have lost confidence in the data presented and consider the conclusions of this manuscript substantially compromised.

